# Let the Girls Romp

**Published:** 1892-04

**Authors:** 


### LET THE GIRLS ROMP.

Most mothers have a dread of romps, so they lecture the girls daily on the proprieties and exhort them to be little ladies. They like to see them very quiet and gentle and as prim as possible. The lot of such children is rather pitiable, for they are deprived of the fun and frolic which they are entitled to. Children—boys and girls—must have exercise to keep them healthy. Deprive them of it and they will fade away like flowers without sunshine.

Running, racing, skipping, climbing—these are the things that strengthen the muscles, expand the chest and build up the nerves. The mild dose of exercise taken in the nursery, with calisthenics or gymnastics, will not invigorate the system like a good romp in the open air. Mothers, therefore, who counsel their little girls to play very quietly, make a mistake. Better the laughing, rosy-cheeked, romping girl than the pale lily-faced one who is called every inch a lady.

The latter rarely breaks things or tears her dresses, or tries her mother's patience as the former does; but after all what do the tearing and breaking amount to? It is not a wise policy to put an old head on young shoulders. Childhood is the time for childish pranks and plays. The girls grow into womanhood soon enough. Let them be children as long as possible, and also give them plenty of fresh air and sunlight.

